# Modelling of Bond Formation during Overprinting of PEEK Laminates

**DOI:** 10.3390/ma17174399

**Published:** 2024-09-06

**Authors:** Simon Hümbert, Jonas Meth, Daniel Fricke, Heinz Voggenreiter

**Affiliations:** German Aerospace Center (DLR), Institute for Structures and Design (BT), 70569 Stuttgart, Germany; meth.jonas@freenet.de (J.M.); daniel.fricke@dlr.de (D.F.);

**Keywords:** additive manufacturing, FFF, material extrusion, overprinting, PEEK, thermoplastic composites, in situ bonding, thermal simulation, diffusion bonding

## Abstract

The rapid technological progress of large-scale CNC (computer numerical control) systems for Screw Extrusion Additive Manufacturing (SEAM) has made the overprinting of composite laminates a much-discussed topic. It offers the potential to efficiently produce functionalised high-performance structures. However, bonding the 3D-printed structure to the laminate has proven to be a critical point. In particular, the bonding mechanisms must be precisely understood and controlled to ensure in situ bonding. This work investigates the applicability of healing models from 3D printing to the overprinting of thermoplastic laminates using semi-crystalline, high-performance material like PEEK (polyether ether ketone). For this purpose, a simulation methodology for predicting the bonding behaviour is developed and tested using experimental data from a previous study. The simulation consists of a transient heat analysis and a diffusion healing model. Using this model, a qualitative prediction of the bond strength could be made by considering the influence of wetting. It was shown that the thermal history of the interface and, in particular, the tolerance of the deposition of the first layer are decisive for in situ bonding. The results show basic requirements for future process and component developments and should further advance the maturation of overprinting.

## 1. Introduction

Material extrusion additive manufacturing of high-performance thermoplastics such as PEEK (polyether ether ketone) or PEI (polyethyleneimine) has become a manufacturing process of choice for aerospace applications in recent years. Some approaches for aerospace applications pursued by the ESA (European Space Agency) are described by Lafont et al. [1,2]. Similarly, there have also been attempts to print CubeSat structures from PEEK [3] or multifunctional satellite structures [4,5]. Further possible applications result from functionalising the 3D printable materials with various fillers [6].

However, there are limits to using 3D printing in load-bearing structures in terms of performance and size. When printing PEEK, high process temperatures, especially nozzle, build chamber and build plate temperatures, are generally required to optimise the mechanical properties [7]. Nevertheless, the mechanical properties remain below those of injection-moulded components [7,8]. 

One approach to overcoming these limitations is to produce hybrid structures by overprinting thermoplastic laminates. The outstanding mechanical properties of continuous fibre-reinforced laminates can be combined with the design freedom and functionalisation of 3D printing. Morales et al. [9] presented a process in which the joint between PA 6 (polyamide 6) laminates and 3D printing is produced in situ during overprinting. Penter et al. [10] and Maier et al. [11] investigated in situ joining in more detail. The implementation of this process in an automated process chain was described by Matkovic et al. [12]. Chauvette et al. [13] describe how overprinting can produce functionalised body structures for aircraft. A similar large-scale approach using SEAM (Screw Extrusion Additive Manufacturing) was used by Atzler et al. [14]. They have shown that the toolpath planning and its tolerances significantly affect the bonding quality during overprinting [15]. Detailed investigations into in situ joining with aerospace-grade material PEEK were carried out by Caprais et al. [16,17] and Hümbert et al. [18,19]. They showed that, as with the printing of PEEK, high process temperatures are also required for in situ joining between PEEK 3D printing and PEEK laminate. Sufficient strength can only be achieved under certain conditions, and stable process control is therefore necessary. The properties of the joint can be traced back to the layer bonding, just like the layer bonding in 3D printing [20]. A deep understanding of this layer structure is, therefore, the basis for the design of the process.

The influence of the process parameters on the mechanical properties of printed PEEK, and thus the layer bonding, has already been investigated in detail. Zanjanijam et al. [7] concluded that both high nozzle temperatures and low gradients between the extruded material and the substrate are crucial. The study by Liaw et al. [20] on bonding strength supports this conclusion. Wang et al. [21] and Yang et al. [22] observe a similar behaviour, also concerning crystallinity.

In addition to the experimental investigation of inter-layer bonding, modelling is a helpful tool for predicting mechanical properties and process design. The modelling includes material extrusion, fibre orientation, residual stresses and inter-layer bonding [23]. Inter-layer bonding is generally divided into three phases: intimate contact, neck growth and molecular diffusion [24]. Intimate contact and neck growth occur in the first material deposition phase. Neck growth was described by Bellehumeur et al. [25] and Kallel et al. [26] using a sintering model, whereby a good correlation between the model and measurement could be demonstrated. In Coogan and Kazmer [27], the intimate contact and, thus, the neck radius were further described by the contact pressure. 

After the neck growth, the inter-layer bonding is formed by diffusion. The diffusion modelling is based mainly on the reptation theory by de Gennes [28,29]. An important conclusion of this theory is that the penetration depth of chains through a plane in an entangled system is proportional to the fourth root of time, $t^{\frac{1}{4}}$. Wool et al. [30,31] have extended this theory to describe molecular diffusion through a polymer–polymer interface under isothermal conditions. For crack healing with instantaneous wetting, the diffusion coefficient *D* is defined by Equation (1), where *σ* is the healed interface strength, *σ_∞_* is the strength of the bulk material, *σ*_0_ is the contribution to strength that is only created by intimate contact, and *K* is a constant.

(1) $$D=\frac{\sigma }{\sigma_{\infty }}=\frac{\sigma_{0}}{\sigma_{\infty }}+\frac{Kt^{\frac{1}{4}}}{\sigma_{\infty }}$$

Jud et al. [32] studied diffusion similarly and were also able to show that diffusion is proportional to $t^{\frac{1}{4}}$ and that diffusion is faster at higher temperatures. They also observed that the diffusion coefficient *D* can be described by the Arrhenius law. Considering these observations, Equation (1) can be reformulated as Equation (2), where *A* is a constant, *E_A_* is the activation energy, and *R* is the universal gas constant.

(2) $$D=\frac{\sigma_{0}}{\sigma_{\infty }}+A\;e^{\frac{{-E}_{A}}{RT\left(t\right)}}\;t^{\frac{1}{4}}$$

Dara and Loos [33] successfully used Equation (2) to describe the diffusion of the polysulfone, and Lee and Springer [34] used it for PEEK 150.

Based on the previous models, Yang and Pitchumani [35] have developed a non-isothermal model for the description of composite manufacturing processes. A temperature-dependent welding time *τ_w_* is used to describe the diffusion in Equation (3).

(3) $$D={\int_{0}^{t}\frac{1}{\tau_{w}(T)}dt}^{\frac{1}{4}}$$

Yang and Pitchumani [35] described *τ_w_* using the fitted Arrhenius equation of Lee and Springer [34] to validate the model. Bastien and Gillespie [36] have shown a similar approach using the Arrhenius law. For 3D printing, Li et al. [37] applied the Arrhenius description to PLA (Polylactic Acid) and Yin et al. [38] to ABS (Acrylonitrile Butadiene Styrene) and TPU (Thermoplastic polyurethane).

An alternative description of *τ_w_*, which has also been successfully applied to 3D printing, can be derived from rheological data as shown by Coogan and Kazmer [27,39] using HIPS (High Impact Polystyrene) or Bartolai et al. [40] and Lepoivre et al. [41] using ABS. Coogan and Kazmer [27] additionally combined the diffusion model with a pressure-driven contact model to take neck growth into account in addition to diffusion and thus improve the accuracy of the prediction. Gilmer [42] has compared the different approaches to *τ_w_* using the example of PEI (Polyetherimide) and shown that the various descriptions predict consistent trends for healing.

In the studies described so far, only amorphous thermoplastics have been investigated, whereby diffusion occurs as long as the interface temperature is above the glass transition temperature *T_g_*. With semi-crystalline thermoplastics such as PEEK, the bonding mechanisms are more complicated, as crystallinity build-up restricts neck growth and healing [24].

To consider this influence, Barocio et al. [43,44] have coupled the diffusion model with the evolution of crystallinity. Diffusion is predicted using a fitted Arrhenius equation stopped after exceeding a critical crystallinity value of 5%. Good predictions for PPS (Polyphenylene sulfide) were made with this model. Consul et al. [45] used a similar approach to compare the healing of PEEK and slow-crystallising PAEK (Polyaryletherketone). It was confirmed that the onset temperature of crystallisation significantly inhibits healing and can be seen as a critical threshold for the healing process. Basgul et al. [46] used a diffusion model for PEEK based on rheological data. Assuming that the diffusion dynamics are negligible when crystallinity is formed, the diffusion was cancelled once the temperature fell below the melting point. Here, too, the experimental results support the model. In all of the modelling, mentioned above, of the healing process of semi-crystalline high-temperature thermoplastics, a contribution by intimate contact is neglected due to the high cooling rates.

The presented diffusion models are based primarily on the thermal history of the interface, which in turn is determined by the process parameters during 3D printing. Various methods are used to predict this thermal history. These range from 1D analytical models, such as those used by Thomas and Rodriguez [47] or Sun et al. [48], to 3D finite element models, such as those used by Zhang et al. [49] or Zhou et al. [50]. The modelling of the 3D-printed path is realised by element death and birth. Brenken et al. [51] have shown that the simulation with the help of the FEM (Finite Element Method) can also be used to calculate the deformation and residual stresses during cooling.

To successfully apply the process of overprinting PEEK laminates to large structures, it is necessary to precisely understand and predict the bonding mechanism between laminate and 3D printing. To this end, this work will investigate to what extent the existing models for describing inter-layer bonding can be applied to in situ joining during overprinting. Firstly, the required material parameters are determined experimentally. The thermal history is then calculated using a 3D FEM heat transfer model. This information is then used in a healing model to calculate the bonding strength. The healing model is used to predict the effect of different process parameters on the bond strength. Both the heat transfer as well as the healing model are validated using experimental data from previous studies [18,19]. 

## 2. Materials and Methods

**Figure 1:**
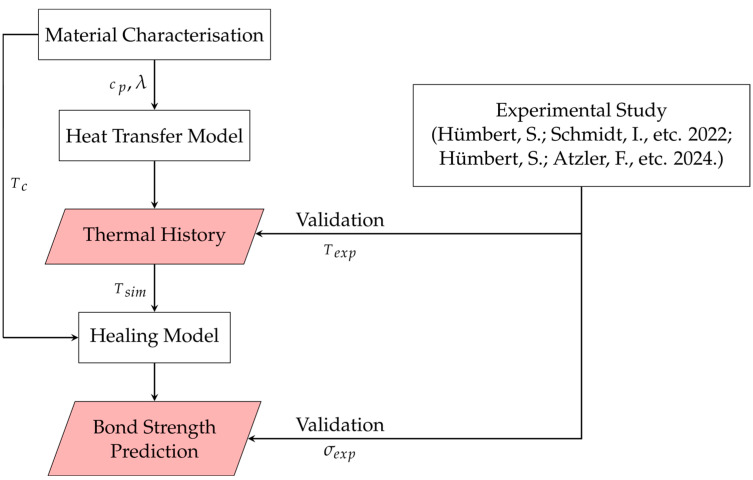
Structure of this study [18,19].

The structure of the study is shown in Figure 1. To carry out the thermal analysis, the thermal properties of the 3D printing material are required, particularly the specific heat capacity *c_p_* and the thermal conductivity *λ*. The crystallisation temperature *T_c_* is particularly important for the healing model. These material data are first determined experimentally. In addition, experimental measurements of the bond strength *σ_exp_* and the interface temperature *T_exp_* are required to validate the simulation models. The methodology and results of these measurements of *σ_exp_* and *T_exp_* were described in previous studies [18,19]. With the help of all these measured values, a FEM analysis of the thermal history and, finally, the healing prediction are carried out.

### 2.1. Materials

The PEEK filament TECAFIL PEEK VX CF30 [52] (Ensinger Plastics, Nufringen, Germany), filled with 30 wt % short carbon fibres, is considered for 3D printing. The high degree of carbon fibre filling increases the stiffness and reduces the thermal expansion of the printed component compared to pure PEEK. This reduces warpage during printing and also reduces the difference in stiffness between the printed component and the overprinted laminate, which is advantageous for the preceding mechanical tests.

**Figure 2:**
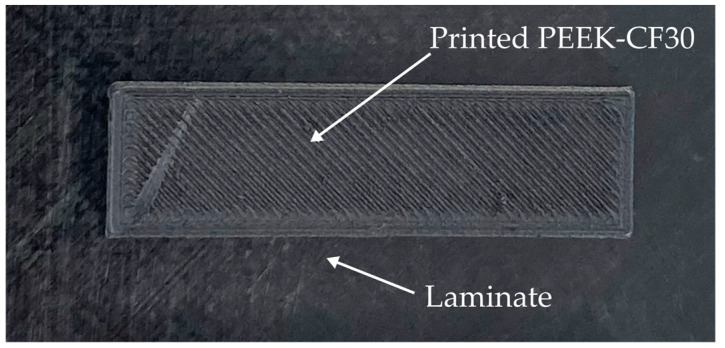
Printed short fibre-reinforced PEEK on continuous fibre-reinforced laminate.

The laminate, which is overprinted, consists of the UD (unidirectional), continuous fibre-reinforced tape Toray Cetex 1200 [53] (Toray Advanced Composites, Morgan Hill, CA, USA) in the configuration [45/0/−45/90]_s_. It is a PEEK tape with 66 wt % carbon fibres. The material properties of both materials are summarised in Table 1. An example of a printed sample of short fibre-reinforced PEEK on the continuous fibre reinforced laminate is shown in Figure 2.

### 2.2. Material Characterisation Methodology

#### 2.2.1. Crystallisation Temperature *T_c_* and Specific Heat Capacity *c_p_*

DSC (Differential scanning calorimetry) was performed using a DSC 214 Polyma (Erich NETZSCH GmbH & Co. Holding KG, Selb, Germany). The measurement was taken for three specimens (10 mg). The measurement program was run under a nitrogen atmosphere and with a heating and cooling rate of 10 °C/min. The samples were heated up and cooled down three times to 420 °C and 20 °C, respectively. The crystallisation temperature *T_c_* was determined as the peak temperature of the crystallisation of the cooling curves. In addition, the melting peak temperature and glass transition mid-temperature were determined by the second and third heating curves.

The specific heat capacity *c_p_* was determined using the same measurement setup. Sapphire was used as the reference material. The *c_p_* curves were determined according to DIN EN ISO 11357-1 [54].

#### 2.2.2. Thermal Conductivity λ

The thermal conductivity was determined using LFA (Laser Flash Analysis). To produce the samples, 50 × 50 × 4 mm^3^ plates were printed, from which round samples with a diameter of 12.6 mm and a thickness of 2.5 mm were then prepared. The LFA 457 MicroFlash measuring device (Erich NETZSCH GmbH & Co. Holding KG, Selb, Germany) was used to measure the thermal diffusivity a of the material. Pyroceram 9606 was used as the reference material. The measurements were carried out from 22 °C to 300 °C. The thermal conductivity *λ* can be calculated from the thermal diffusivity by Equation (4)

(4) $$\lambda \left(T\right)=a\left(T\right)\;c_{p}\left(T\right)\;\rho \left(T\right),$$

where *a* is the measured thermal diffusivity, *c_p_* is the measured specific thermal capacity, and *ρ* is the density. *ρ* is assumed to be approximately constant at 1.38 g/cm^3^.

### 2.3. Heat Transfer Model Methodology

**Figure 3:**
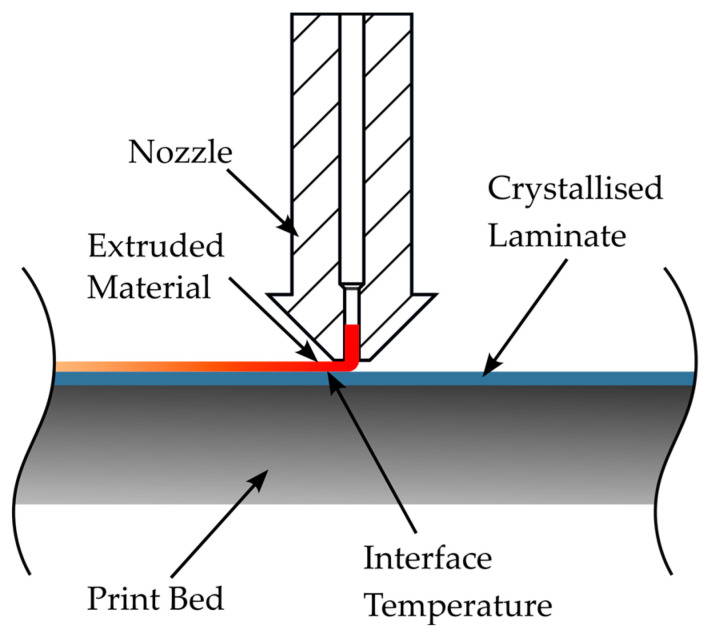
Schematic of material discharge during overprinting of the laminates for the production of mechanical specimens.

The heat transfer model for calculating the thermal history of the interface between the 3D-printed component and the laminate was implemented using a FEM model in the software Ansys Mechanical (Ansys, Inc., Canonsburg, PA, USA), version 2023 R2. In the context of this work, the simulation results are to be evaluated using measured values from previous studies [18,19]. For this reason, the print setup for the mechanical test specimens was modelled in the simulation. The samples were initially printed with the GEWO HTP 260 (GEWO3D, Wörth, Germany) filament printer, which has a heated print bed and an enclosed, heated build chamber. The basic structure of the material deposition to be modelled is shown in Figure 3. 

In the model, the material deposition is modelled by element activation. The element activation is time-step-controlled by an Ansys APDL (Ansys Parametric Design Language) script. The print direction per layer can be freely selected. In this work, an infill with an orientation of ±45° is used as in the reference tests. In each time step, a new element is activated and the node temperatures of the element are set to the extrusion temperature for the duration of the time step. In the time step after depositing, the node temperatures are released, and the material can cool down according to the ambient conditions. 

**Figure 4:**
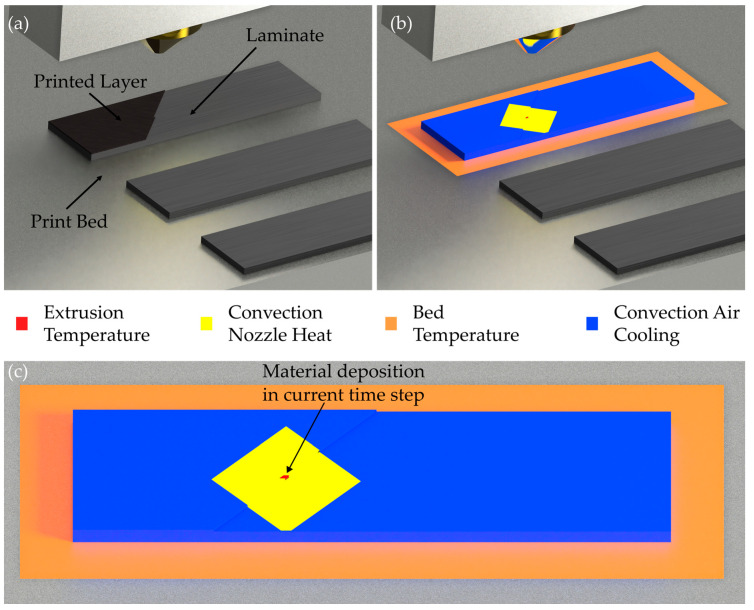
Geometry of simulated specimens (**a**) and loads and boundary conditions of the simulation (**b**); detail view of loads and boundary conditions (**c**).

The investigated process parameters are represented by boundary conditions and convection loads. The nozzle or extrusion temperature is represented by the constraint of the newly activated element. The print bed temperature is also modelled by a boundary condition. For this purpose, the temperature of the underside of the laminate, which rests on the print bed, is limited to the print bed temperature. Convection cooling is applied to the free surfaces of the laminate and the already-printed material in accordance with the chamber temperature. The convection coefficient was calculated from the estimated flow conditions with a value of 300 W/(m^2^ K). Finally, an additional convection load is applied under the nozzle to map the influence of the heated nozzle directly above the sample. The temperature of the convection load corresponds to the nozzle temperature as a simplifying assumption. The geometry of the simulated specimens is shown in Figure 4a. The loads and boundary conditions are shown in Figure 4b,c.

**Figure 5:**
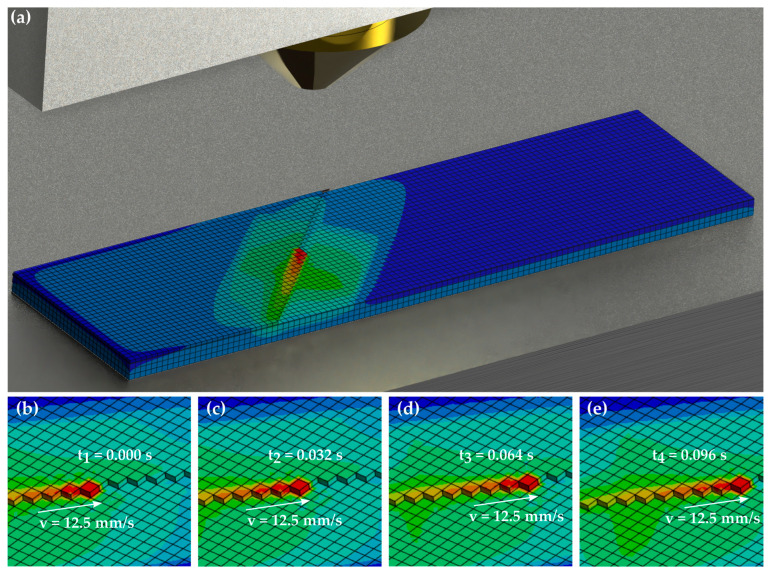
Resulting temperature distribution (**a**) and scheme of element activation (**b**–**e**).

The element size was selected so that one element approximately covers the outlet surface of the nozzle. As a 0.4 mm nozzle was used for printing in this case, an element size of 0.4 × 0.4 mm^2^ was selected. The maximum thickness of the elements is 0.4 mm. Since the layer thickness of the printed layer is thinner than 0.4 mm, the element thickness in the printed layers is the corresponding layer height. The layer heights are varied in this work, whereby the element thickness of the printed layers changes accordingly. The length of the time steps was selected so that the elements are activated according to the printing speed. This means that the nozzle is also applied to the model at the correct printing speed. With an element size of 0.4 mm and a printing speed of 12.5 mm/s, this means a time step of 0.032 s. Figure 5a shows the meshed specimen during element activation, and Figure 5b–e show a scheme of the element activation.

**Figure 6:**
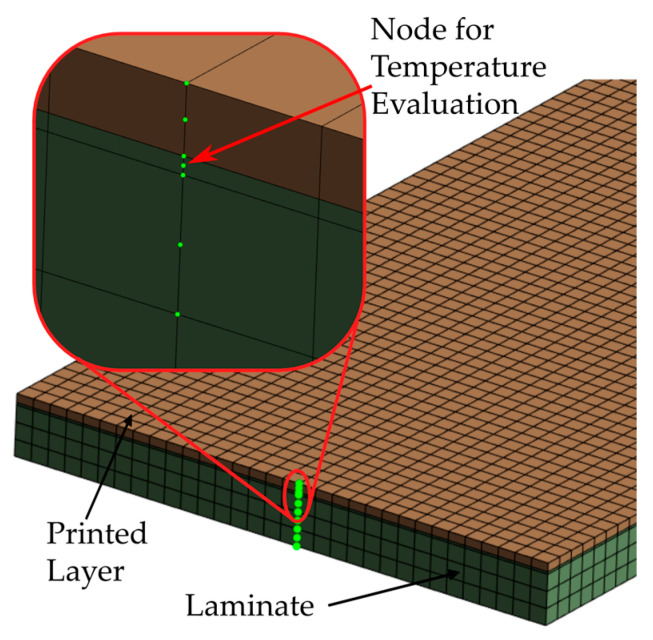
Mesh setup for the interface temperature evaluation.

For the subsequent calculation of the bonding strength, the temperature profile in the interface between the printed material and the laminate is required. Evaluating a node directly in the interface between the newly activated element and the laminate does not make sense in this model, as these node temperatures inevitably correspond to the extrusion temperature due to fixing the node temperatures during deposition. A more sensible approach is to determine the temperature after a minimum penetration depth into the laminate to also consider the energy input into the laminate. For this purpose, a thin element plane with a thickness of 0.05 mm was inserted at the top of the laminate. As with all other elements, these are quadratic elements, and the interface temperature was evaluated at the centre node, i.e., at a distance of 0.025 mm from the surface. The setup for the temperature evaluation is shown in Figure 6.

The density, specific heat capacity and thermal conductivity are required for the respective material model in the thermal simulation. While the density and heat capacity are direction-independent, the thermal conductivity of fibre-reinforced plastics is generally anisotropic. Material data are available in the literature for the PEEK UD tapes used to make the laminate. As suggested and successfully used by [55], the data from [56] were used in this work. The quasi-isotropic properties of the laminate were calculated from the data of the tapes according to the model of [57]. The laminate is described by an in-plane and an out-of-plane value due to the quasi-isotropic fibre. The material properties of the laminate used are summarised in Table 2. The material parameters required to describe the short-fibre-reinforced PEEK-CF 3D printing material are determined experimentally as part of this work. A slight anisotropy is also to be expected for 3D printing material. However, since only the first layer is relevant in this model and the thermal conductivity perpendicular to the printing direction is dominant for the heat input into the interface, the model is simplified and assumed to be an isotropic material with the thermal conductivity perpendicular to the printing direction.

Using this model, a total of 15 parameter variations were calculated, whereby the nozzle temperature, the print bed temperature, the build chamber temperature and the layer thickness of the first layer were varied. The build chamber temperature is always 10 degrees below the print bed temperature. All other process parameters were kept constant. Previous analysis has shown that the extruded material’s melt temperature in the given range is approximately 54.6 °C below the set nozzle temperature. This difference was taken into account accordingly in the simulation. The parameter ranges are summarised in Table 3. A detailed list of all parameters can be found in Table A1 in Appendix A.

### 2.4. Healing Model Methodology

As already described, the layer bonding in 3D printing is primarily driven by intimate contact, neck growth and diffusion. Similar behaviour is expected in the bond between the printed material and the laminate during overprinting, as the same thermoplastic is present as a matrix in both the laminate and the 3D printing filament. Various investigations also suggest that the *σ*_0_ component due to intimate contact, as suggested by Wool et al. in Equation (2), is negligible [40,41,44,45,46,58,59,60]. This agrees with the observations of the previous experiments on overprinting [18,19], in which pure wetting does not lead to any measurable bond strength.

Assuming that the bonding is mainly caused by diffusion, the non-isothermal healing model presented by Yang and Pitchumani [35], as in Equation (3), is used to predict the strength between the 3D-printed material and the laminate. The fitted Arrhenius equation by Lee and Springer [34] is used to describe *τ_w_*. This model was adapted for PEEK 150, which is also used for 3D printing in this study. In addition, Yang and Pitchumani [35] have already successfully tested the model in their own work.

**Figure 7:**
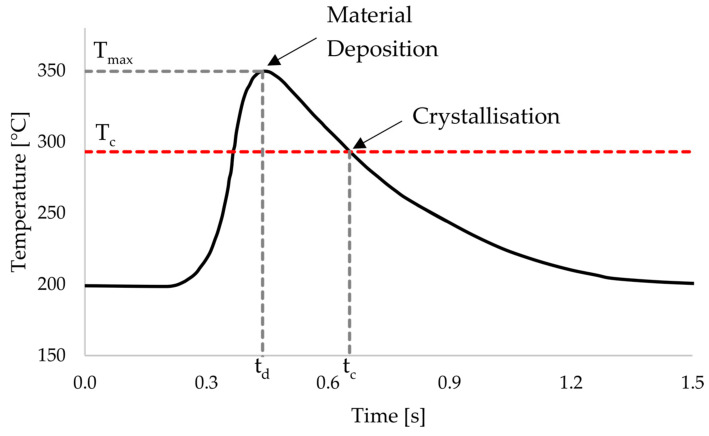
Schematic representation of the temperature curve of the interface.

One difference between traditional 3D printing and the overprinting of laminates is that in the case of overprinting, the temperature of the substrate cannot be influenced by the print settings. Accordingly, the thermoplastic in the laminate is already crystallised, and the energy input to enable bonding in the interface must take place entirely through the newly printed layer. Assuming that diffusion mainly occurs in the amorphous state and is so strongly inhibited by crystallisation that a contribution in the crystalline state is negligible, this means that the interface temperature must be brought above the melting temperature of PEEK during overprinting. In addition, diffusion only occurs in the time interval between the deposition time *t_d_* and the time of crystallisation time *t_c_* during the cooling phase. This time interval is shown schematically in Figure 7. 

Taking into account the assumptions for *τ_w_* and the time interval for diffusion, Equation (3) results in Equation (5), which is used below to calculate the strength between 3D printing and laminate. As no fibres can run through the interface layer between the laminate and 3D print, the fully healed bond strength *σ_∞_* is assumed to be the strength of the used PEEK matrix. The average strength of printed PEEK is specified by the manufacturer as 90 MPa [61]. Therefore, *σ_∞_* is assumed to be 90 MPa for this work.

(5) $$D=\frac{\sigma }{\sigma_{\infty }}={\int_{t_{d}}^{t_{c}}{\left(\frac{1}{44.1}\;e^{\frac{3810}{T\left(t\right)}}\right)}^{-4}dt}^{\frac{1}{4}}$$

Using Equation (5) and the temperature curve from the thermal calculation, the bond strength is calculated for all parameter variations in Table A1 in Appendix A. The prediction method is evaluated by comparing these calculated strengths with the measured values.

### 2.5. Statistics

To compare the simulation results with the experimental values, the results were integrated into the statistical evaluation of the experiments. The assessment was carried out in the DoE software Design Expert, version 23.1.5 (Stat-Ease, Inc., Minneapolis, MN, USA). Surface response models were created with this software using the simulation results and compared with the models of the experimental values. The 95% confidence interval was specified for all models. In addition, statistical correlations between the experiment and simulation were determined and tested for significance using ANOVA (analysis of variance). The correlations are indicated by the Pearson correlation factor r. Correlations with a *p*-value below 0.05 are considered significant.

## 3. Results

This section presents the results of the material characterisation, the thermal simulation, and the strength prediction. The simulation results are compared with the experimental results to be able to evaluate the simulation methodology.

### 3.1. Material Characterisation Results

**Figure 8:**
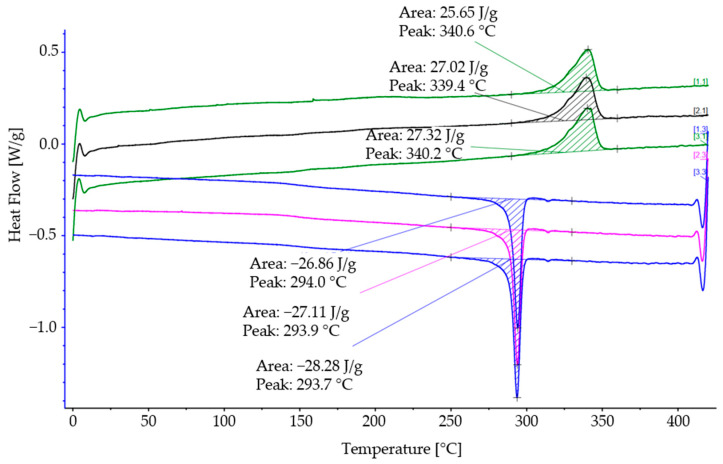
Heating and cooling DSC curves of one specimen of PEEK-CF filament.

DSC measurements were carried out on three samples of the PEEK-CF filament. As an example, the three heating and cooling DSC curves of a sample are shown in Figure 8. The three samples showed very uniform results. On average, the peak melting temperature *T_m_* is 340 °C during heating. The peak crystallisation temperature *T_c_* is, on average, 294 °C.

**Figure 9:**
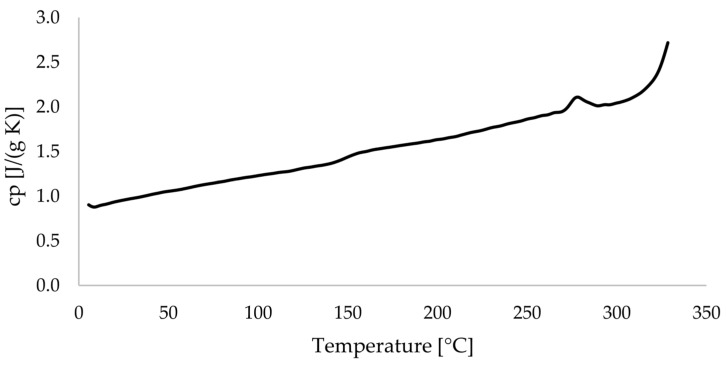
Averaged *c_p_* curve of PEEK-CF filament.

The specific heat capacity *c_p_* was determined based on the heating curves. The averaged *c_p_* curve is shown in Figure 9.

**Figure 10:**
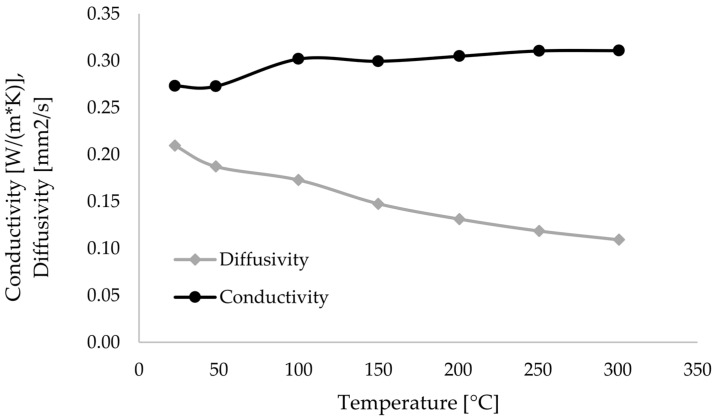
Measured thermal diffusivity and conductivity of PEEK-CF filament.

The thermal conductivity was determined using LFA. Figure 10 shows the average course of the thermal diffusivity and the resulting thermal conductivity.

### 3.2. Heat Transfer Model Results

**Figure 11:**
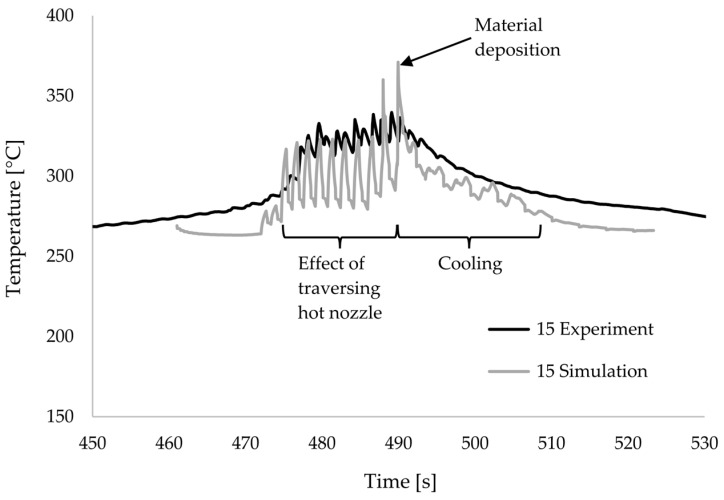
Measured and simulated temperature curves of run 15.

The heat transfer model was used to simulate the temperature curve of the interface between laminate and 3D-printed material from the 15 experimental printing configurations. The measured and simulated temperature curves from run 15 are shown as examples in Figure 11. Both in the experiment and in the simulation, the temperature curve can be divided into three phases. In the first phase, the measuring point is heated up by the approaching nozzle. The movement of the nozzle over the measuring point results in a sawtooth-like temperature curve with several peaks. In the second phase, the material is deposited. This is recognisable as a temperature maximum. In the subsequent third phase, the material cools down. The influence of the removing nozzle is still recognisable, albeit with significantly smaller peaks, as the measuring point is now covered with a layer of material. The comparisons of the temperature curves in the other 14 test runs show consistent behaviour and deviations of a comparable magnitude.

It is noticeable that the peaks with the approaching nozzle and in the cooling phase are much more pronounced in the simulation than in the measurement. The peak of the maximum temperature is also higher in the simulation than in the measurement. On the other hand, the cooling rate after the peaks and after material deposition is also higher in the simulation than in the measurement. This observation occurs in all 15 configurations.

**Figure 12:**
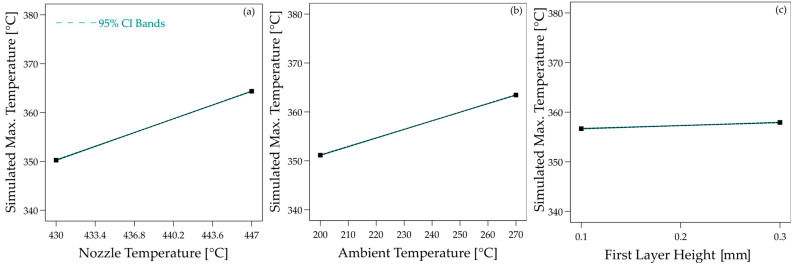
Effect of the process parameters ((**a**): Nozzle Temperature); (**b**): Ambient Temperature; (**c**): First Layer Height) on the simulated maximum temperature.

Like the experiments, the effects of the process parameters on the simulated maximum temperature were analysed using a statistical surface response model. The results are shown in Figure 12.

The calculated maximum temperatures can be mapped very well using the statistical model, which is reflected in a very narrow confidence interval and the statistical significance (*p* < 0.0001) of all three effects. However, the strength of the effects is only notable for the nozzle temperature (*r* = 0.790) and the ambient temperature (*r* = 0.729). With a Pearson correlation of *r* = 0.097, the first layer height has a minimal effect on the maximum temperature.

**Figure 13:**
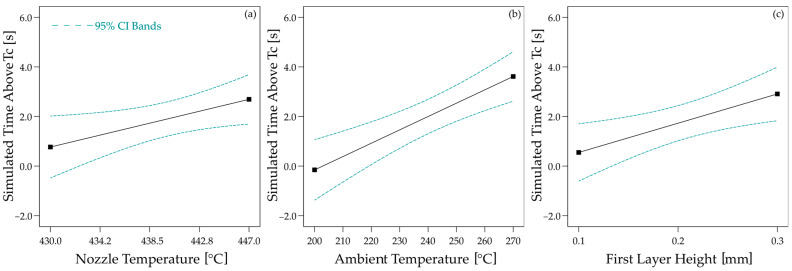
Effects of process parameters ((**a**): Nozzle Temperature); (**b**): Ambient Temperature; (**c**): First Layer Height) on simulated time above crystallisation temperature *T_c_*.

The essential key figure of the time above the crystallisation temperature *T_c_* shows similar correlations as the maximum temperature. Here, too, all three effects are statistically significant, although the effect of the nozzle temperature (*p* = 0.0349, *r* = 0.443) is less pronounced than for the maximum temperature. The layer thickness of the first layer, on the other hand, has a stronger influence (*p* = 0.0121, *r* = 0.435). The ambient temperature again has the strongest influence on the time over *T_c_* (*p* = 0005, *r* = 0.725). The effect plots are shown in Figure 13.

Table 4 compares the effects of the process parameters on the measured temperature curves with those on the simulated temperature curves. The calculated effects largely match the experimental results in terms of quality. The influence of the nozzle temperature on the maximum temperature and the time above *T_c_* is predicted to be higher in the simulation than was determined in the measurement. However, it should be noted that these two effects from the experiment are not statistically significant. The simulative prediction also deviates slightly from the experiment for the effect of the first layer height, but here, too, the experimental values are not statistically significant. The quantitative agreement is very good for all significant effects.

**Figure 14:**
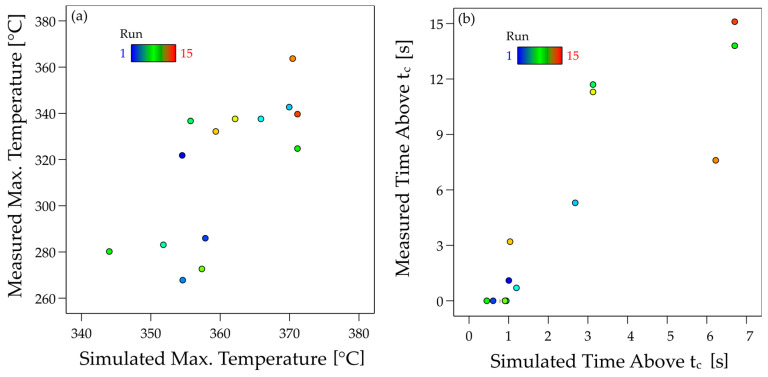
Comparison of simulated vs. measured maximum temperature (**a**) and simulated vs. measured time above *T_c_* (**b**).

A quantitative comparison between the experiment and simulation is shown in Figure 14, in which the individual values of the maximum temperature (a) and time over *T_c_* (b) are plotted against each other. The correlation between simulation and experiment is recognisable for the maximum temperature. This is also statistically verifiable with *r* = 0.721. It is noticeable that the maximum temperatures in the simulation are higher on average than in the experiment. The correlation between simulation and experiment is much stronger for the time above *T_c_* with *r* = 0.881. Here, however, the predicted values of the simulation are, on average, lower than the measured values.

### 3.3. Healing Model Results

**Figure 15:**
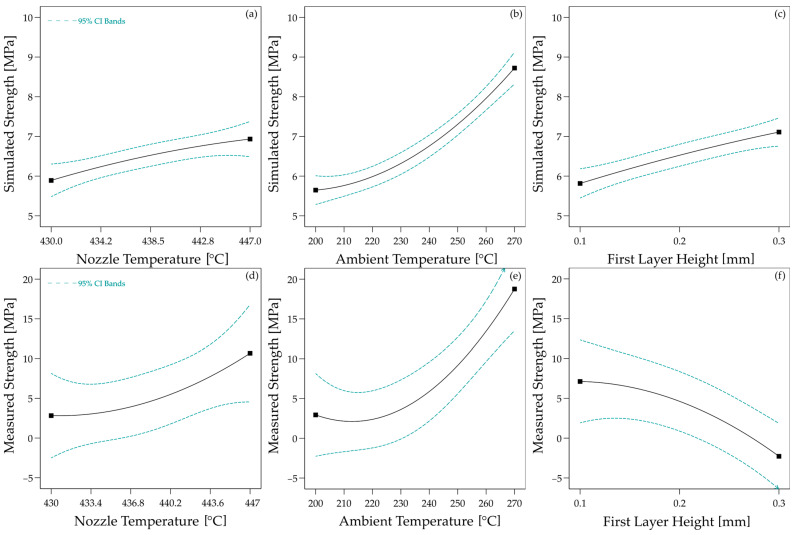
Effects of the process parameters on the simulated bond strength ((**a**): Effect of Nozzle Temperature; (**b**): Effect of Ambient Temperature; (**c**): Effect of First Layer Height) and the meas-ured bond strength [19] ((**d**): Effect of Nozzle Temperature; (**e**): Effect of Ambient Temperature; (**f**): Effect of First Layer Height).

Based on the simulated temperature curves, the predicted strength between the 3D-printed material and the laminate was calculated for all parameter configurations using Equation (5). As with the heat transfer simulation results, the calculated strengths were incorporated into the statistical model from the experimental study to compare simulation and experiment. The effects determined in this way are shown in Figure 15a–c. For direct comparability with the experiment, the effects on the experimentally determined strengths are shown in Figure 15d–f.

All three effects are also significant for the calculated strength (nozzle temperature: *p* = 0.0007; ambient temperature: *p* < 0.0001; first layer height: *p* = 0.0002). Concerning the nozzle temperature, the effects also qualitatively match the effects determined experimentally. However, the effect of the ambient temperature, in particular, is overestimated in the simulation. The statistical parameters are summarised in Table 5. For the first layer height, the simulation even predicts the opposite behaviour to that observed in the experiment. In the simulation, higher layer thicknesses lead to higher strengths. Since the simulation has also calculated higher maximum temperatures and longer times above *T_c_* for higher layer thicknesses and the temperature curve is the only input of the healing model, no other behaviour is to be expected. In the experiment, however, higher layer thicknesses lead to lower strengths.

The results suggest that the temperature curve is not the only factor influencing bond formation. In the experimental study [19], it was already established that not only the set value can be considered when analysing the first layer height. Here, it was shown that the accuracy of the calibration of the layer height on the printer must also be taken into account, as the tolerance of the machine used already leads to significant scattering of the experimental results. To compensate for this uncertainty, the height of the first layer was measured optically for each individual specimen.

It is reasonable to assume that a deviation between the target and actual values of the first layer height also influences the bond formation. In particular, if the actual distance between the nozzle and the laminate is greater than set, this leads to underextrusion and, thus, to incomplete wetting of the laminate. The negative influence of underextrusion on bond formation has already been shown in a previous study on large-scale manufacturing [15]. Coogan and Kazmer [27] investigated surface wetting in 3D printing and also found that, depending on the process parameters, the surface of the substrate is not always completely wetted, which can lead to a reduction in the effective bonding area. Their broad study included the reduced bonding area as a linear factor in the healing Equation.

Following these studies, the healing model from Equation (5) used here is also adjusted by a linear wetting factor as in Equation (6).

(6) $$D=\frac{\sigma }{\sigma_{\infty }}={c_{wetting}\int_{t_{d}}^{t_{c}}{\left(\frac{1}{44.1}\;e^{\frac{3810}{T\left(t\right)}}\right)}^{-4}dt}^{\frac{1}{4}}$$

**Figure 16:**
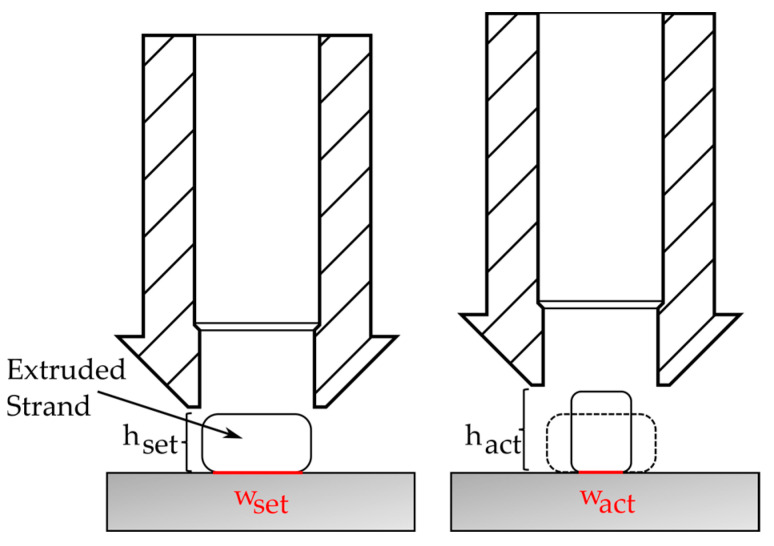
Calculation of the deviation of the contact width from the measured layer heights.

The wetting factor *c_wetting_* is intended to represent the deviation of the actual contact width *w_act_* from the set contact width *w_set_*, which in this model would correspond to the nozzle diameter. Since the ratio of the set layer height *h_set_* to the actual layer height *h_act_* is known from the measurements of the specimens, the deviation of the contact width is derived from this in a simplified manner. The procedure is illustrated in Figure 16.

When calculating *c_wetting_*, it is assumed that the extruded strand of material is approximately rectangular and that the cross-section of the extruded strand remains constant when the layer height is changed. This assumption should be largely correct, as the extruded mass flow in the toolpath planning depends solely on the set layer height, the nozzle diameter and the printing speed. There is no mechanical coupling between the layer and the filament feed. Under these assumptions, *c_wetting_* is obtained as in Equation (7). The prediction of the bond strength is recalculated using Equation (8).

(7) $$c_{wetting}=\frac{w_{act}}{w_{set}}=\frac{h_{set}}{h_{act}}$$

(8) $$D=\frac{\sigma }{\sigma_{\infty }}={\frac{h_{set}}{h_{act}}\int_{t_{d}}^{t_{c}}{\left(\frac{1}{44.1}\;e^{\frac{3810}{T\left(t\right)}}\right)}^{-4}dt}^{\frac{1}{4}}$$

**Figure 17:**
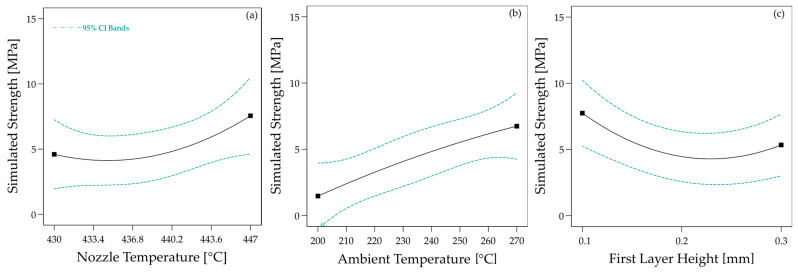
Effects of the process parameters ((**a**): Nozzle Temperature); (**b**): Ambient Temperature; (**c**): First Layer Height) on the simulated bond strength using the wetting factor *c_wetting_*.

By including the layer height of each individual specimen, the initial 15 calculated cases became 70 strength values. The layer heights from run 6 could not be considered as the measurement failed. Accordingly, run 6 was not included in the evaluation. The complete list of measured first layer heights per run is summarised in the Table A2 in the Appendix A. The result of the calculated effects is shown in Figure 17.

The magnitude of the predicted effects is in much better agreement with the measured effects. Above all, the predicted effect of the first layer height now matches the measurements qualitatively (see Table 5). However, the certainty of the prediction has also decreased. This is expressed by a wide confidence band and higher *p*-values (nozzle temperature: *p* = 0.0300; ambient temperature: *p* < 0.0001; first layer height: *p* = 0.0622). Although the predicted effect of the first layer height now agrees better with the experiment, it is not statistically significant.

**Figure 18:**
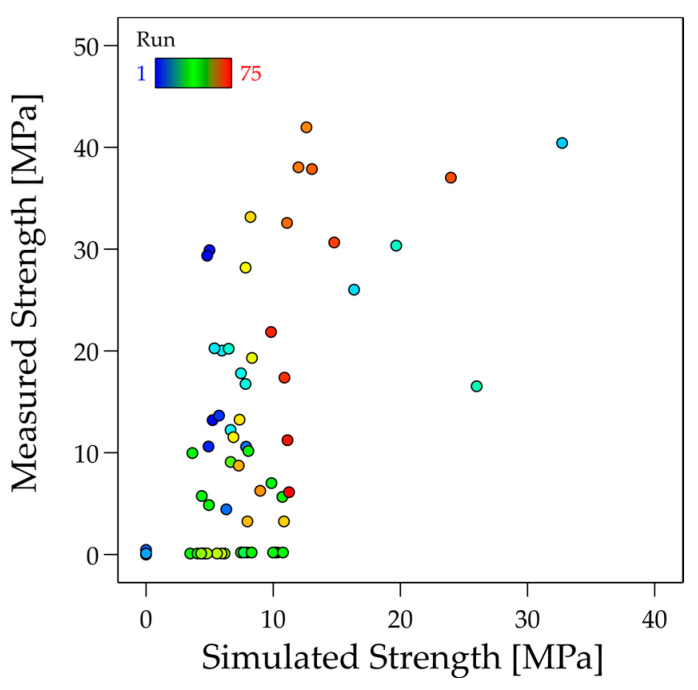
Comparison of simulated vs. measured strength using *c_wetting_*.

A quantitative comparison of the measured values and simulation is shown in Figure 18. Although a common trend is recognisable, the high scattering of the measured values and the effect of the scattering of the layer height on the simulation are evident.

## 4. Discussion

The characteristic properties of the interface’s temperature curves could be mapped well in the transient thermal heat transfer calculation. The influence of the toolpath planning, the moving hot nozzle, and the material discharge behaved as in the measurements. The cooling behaviour is also qualitatively consistent. Accordingly, the effects of the process parameters on the important parameters were also reproduced in qualitative agreement with the test results.

Overall, however, both the maximum temperatures and the cooling rates were predicted to be higher than those measured in the test. This can be seen in the effect plots shown, but also in the direct comparison of the temperature curves as in Figure 11. A very good agreement can be found in the literature for simple single-wall geometries. Sun et al. [48] compared various analytical models and achieved good results. Lepoivre et al. [41] were able to map the temperature curve of single-wall geometries very accurately in the multiphysics software COMSOL. However, with more complex 3D models, deviations often occur as in this work. The FEM model by Yin et al. [38] also predicted significantly higher temperature peaks than measured. Basgul et al. [46] were able to show that these deviations can shift over several layers.

One possible cause of deviation in this work may be the type of temperature measurement used in the experiment. Type K thermocouples with a diameter of 0.25 mm were used. The response time of these thermocouples is very short but can still have a small influence. The embedding of the elements in the laminate is more decisive. In the simulation, the measuring point is 0.025 mm below the surface, and there is a perfect heat transfer to the temperature evaluation node. In the measurement, the thickness of the matrix layer on the thermocouple can vary slightly. The heat transfer from the matrix to the thermocouple is probably not ideal, either. In addition, the heat transfer in the vertical direction between the 3D print and the laminate is also ideal in the simulation. In reality, porosity can occur here, and incomplete wetting further impairs heat transfer, which presumably results in poorer heat transfer in the vertical direction compared to the horizontal direction. In the measurements [19], a porosity of 15–20% was found in the 3D-printed material. In the boundary layer, the porosity is significantly lower, but the exact influence is not known at the time of this work.

Another cause may lie in the loads and boundary conditions of the simulation. The heat transfer coefficient of the convection conditions was calculated from the estimated flow conditions. The heat transfer from the 3D-printed layer to the laminate is also not exactly known. Certain deviations may occur here, especially with regard to the cooling rates.

For a final validation of the heat transfer model, a more detailed campaign is necessary. In particular, the sources of error from the measurement campaign, especially the embedding of the thermocouple and the heat transfer from the laminate to the thermocouple, must be better characterised. In addition, a simpler geometry, e.g., single-wall geometries, should be selected for these tests to avoid superimposed effects from rapid tool movements above the measuring point. In addition, the limiting heat transfer between printed boundary layers should be investigated in more detail.

The healing model initially predicted the effects of the process parameters only inadequately. The results showed that the interface temperature curve was insufficient to simulate the bonding mechanisms when overprinting the laminates in the experimental study. Lee and Springer [34], as well as Yang and Pitchumani [35], came to good agreement with their experiments using the same approach for the welding time *τ_w_* for PEEK. However, both studies used a manufacturing process with considerable consolidation pressure, which presumably makes the influence of wetting negligible. With 3D printing, a few authors, such as Li et al. [37], have successfully predicted the strength with reasonable accuracy without considering the actual wetted area. However, these are also thermoplastics that exhibit good wetting behaviour, especially PLA. Other authors consider the wetting effects intrinsically when the model is fitted by experiments in the same process, as seen in Barocio et al. [44] or Yin et al. [38].

In this work, a linear coefficient was introduced that considers the ratio of the ideal wetted area to the actual wetted area in the model to improve the prediction. A similar approach was used by Consul et al. [45] for PEEK, where the factor mainly takes porosity into account. Coogan and Kazmer [27] have shown that the consolidation pressure due to extrusion significantly influences the actual wetted area and have described this using a wetting factor. This approach has led to very good results. As this consolidation pressure is primarily influenced in this work by the fluctuations in the layer height and the associated over- or underextrusion, the approach seems suitable for describing the effects.

The measured strengths from the experimental study are subject to a high degree of scatter, making final validation of the healing model difficult. In addition, the scatter is projected onto the simulated strengths using measured values for the wetting coefficient. The measurement of the layer thicknesses is also subject to a certain degree of measurement inaccuracy, which is not precisely known here. Overall, the calculated strengths are lower than the measured strengths. This may be because the welding time *τ_w_* model is not optimised for the specific material, but it may also be an effect of the high cooling rates from the heat transfer model. Some factors are also not taken into account in the model. According to the manufacturer, the tapes used in the laminate should have a matrix-rich surface, but there can still be contact between the fibre and the 3D printing material, which is not modelled. Likewise, the influence of the short fibres and the porosity in the 3D printing material is not considered.

To improve the model, a separate campaign to fit *τ_w_* should be carried out in future studies. At the same time, the influence of process inaccuracies should be reduced. The precision with which the first layer height can be set depends heavily on the printer used. By using a newer-generation printer, the scatter can probably be greatly reduced. In addition, the exact nature of the interface must be better determined. This includes both the proportion of fibres and the proportion of porosity. A contact model such as that developed by Coogan and Kazmer [27] can make a useful contribution.

Nevertheless, important conclusions for process development can be derived from the results. In particular, the accuracy of the first layer is a critical factor when large laminates are to be printed with robot-based printers. The positioning accuracy of the robots is often in the same order of magnitude as the layer height. Accordingly, it will be necessary to use very precise, highly calibrated robots. Likewise, the tolerances of large laminates are often greater than the layer thickness. Therefore, it will be necessary to capture the real geometry very precisely and carry out the path planning on this real geometry. These findings are consistent with the experimental results of Atzler et al. [15].

## 5. Conclusions

This work investigated the applicability of diffusion healing for the prediction of in situ bonding during the overprinting of thermoplastic laminates by material extrusion. The work aimed to determine and describe the most important bonding mechanisms to identify important requirements for developing a stable process for large structures. The basis for the healing model is the thermal history in the interface between printed material and laminate. To determine this thermal history, a FEM model was set up to map the 3D printing toolpath. The temperature history was compared with measured values from a previous study, and the most important effects of the printing process on the temperature history were successfully predicted.

When calculating the bonding strength, it was shown that the thermal history alone is insufficient to describe the bonding. A strong influence of the contact pressure due to over- or underextrusion was shown, which strongly affects the bond strength. This effect was considered by an additional factor, whereby the model could finally depict the relationships between process and strength. Although some aspects remain open for holistic modelling, important conclusions can be drawn for further process development:

- Diffusion healing, driven by thermal history, is the main contributor to creating sufficient in situ bonding when overprinting laminates.
- To enable diffusion, the surface of the laminate must be heated above the melt temperature and kept above the crystallisation temperature. Due to the high cooling rates, the interface is usually not fully healed.
- The distance between the laminate and the nozzle must be calibrated very precisely to wet the laminate completely. Any deviation in the first layer height must be considered during bonding.
- For the overprinting of large structures, this means that the toolpath planning must be carried out on the measured real geometry of the laminate, and a robot or portal must be used with very high precision.

Overall, the work highlights some challenges for the process and its modelling, which need to be solved both technologically and through further studies.

## Figures and Tables

**Table 1:** Material properties of PEEK-CF [52] filament and PEEK UD tapes [53].

Property	PEEK-CF30 Filament	Cetex 1200 UD-Tape
Density [g/cm^3^]	1.38	1.59
Young’s modulus [MPa]	17,500	159,000
Ultimate strength [MPa]	190	3100
Glass transition temperature [°C]	143	143
Melting temperature [°C]	343	343
Service temperature [°C]	260	-

**Table 2:** Thermal material properties of the PEEK-CF laminate (adapted from [56]).

Temperature	Specific Heat Capacity	Thermal Conductivity [W/(m K)]	
[°C]	[J/(g K)]	In-Plane	Out-of-Plane
0	0.80	2.37	0.42
50	0.93	3.09	0.52
100	1.04	3.44	0.60
150	1.26	3.98	0.70
200	1.30	3.98	0.70
250	1.40	4.10	0.70
300	1.55	4.50	0.75
350	1.65	4.51	0.68
400	1.70	4.62	0.65

**Table 3:** Parameter ranges of the performed simulations.

**Process Parameter**	**Range**
Deposition Temperature [°C]	375.4–392.4
Print Bed Temperature [°C]	200–270
Chamber Temperature [°C]	200–260
Speed [mm/s]	12.5
First Layer Height [mm]	0.1–0.3
Nozzle Diameter [mm]	0.4
Layer Height Other Layers [mm]	0.2
Infill Raster [°]	±45

**Table 4:** Comparison of the effects of the process parameters on the measured temperature curves [19] and the simulated temperature curves.

Parameter	Nozzle Temperature		Ambient Temperature		First Layer Height	
	Significant	Correlation	Significant	Correlation	Significant	Correlation
Simulated Max. Temperature	yes	0.790	yes	0.729	yes	0.097
Measured Max. Temperature	no	0.335	yes	0.879	no	−0.083
Simulated Time above *T_c_*	yes	0.443	yes	0.725	yes	0.435
Measured Time above *T_c_*	no	0.263	yes	0.757	yes	0.419

**Table 5:** Comparison of the effects of the process parameters on the measured strength [19] and the simulated strength.

Parameter	Nozzle Temperature		Ambient Temperature		First Layer Height	
	Significant	Correlation	Significant	Correlation	Significant	Correlation
Simulated Strength, no wetting	yes	0.451	yes	0.849	yes	0.399
Simulated Strength, wetting	yes	0.394	yes	0.449	no	−0.403
Measured Strength	yes	0.325	yes	0.567	yes	−0.597

## Data Availability

The data presented in this study are available on request from the corresponding author.

## Appendix A

**Table A1:** Parameter variation of the reference tests.

Run	Nozzle Temperature [°C]	Ambient Temperature [°C]	Layer Height [mm]
1	430	262	0.1
2	439	239	0.1
3	440	213	0.2
4	447	270	0.1
5	447	244	0.2
6	434	221	0.3
7	430	263	0.3
8	430	200	0.2
9	447	270	0.3
10	445	200	0.1
11	445	200	0.3
12	437	270	0.2
C13	440	240	0.2
C14	447	270	0.2
C15	447	270	0.3

**Table A2:** Measured first layer height of all reference specimens.

Run	Nozzle Temperature [°C]	Ambient Temperature [°C]	Set First Layer Height [mm]	Measured First Layer Height [mm]
1-1	430	262	0.1	0.124
1-2				0.135
1-3				0.130
1-4				0.132
1-5				0.113
2-1	439	239	0.1	0.210
2-2				0.192
2-3				0.203
2-4				0.096
2-5				0.077
3-1	440	213	0.2	0.269
3-2				0.264
3-3				0.338
3-4				0.277
3-5				0.277
4-1	447	270	0.1	0.010
4-2				0.050
4-3				0.152
4-4				0.137
4-5				0.123
5-1	447	244	0.2	0.195
5-2				0.186
5-3				0.224
5-4				0.074
5-5				0.056
6	434	221	0.3	N/A
7-1	430	263	0.3	0.309
7-2				0.321
7-3				0.330
7-4				0.250
7-5				0.230
8-1	430	200	0.2	0.221
8-2				0.221
8-3				0.253
8-4				0.294
8-5				0.228
9-1	447	270	0.3	0.295
9-2				0.283
9-3				0.367
9-4				0.305
9-5				0.300
10-1	445	200	0.1	0.123
10-2				0.109
10-3				0.148
10-4				0.067
10-5				0.081
11-1	445	200	0.3	0.300
11-2				0.428
11-3				0.386
11-4				0.332
11-5				0.312
12-1	437	270	0.2	0.203
12-2				0.216
12-3				0.246
12-4				0.230
12-5				0.206
13-1	440	240	0.2	0.125
13-2				0.170
13-3				0.186
13-4				0.170
13-5				0.151
14-1	447	270	0.2	0.152
14-2				0.160
14-3				0.173
14-4				0.147
14-5				0.080
15-1	447	270	0.3	0.206
15-2				0.280
15-3				0.310
15-4				0.274
15-5				0.271
